# Pollinator activity and flowering in agricultural weeds in Sweden

**DOI:** 10.1002/ece3.11725

**Published:** 2024-07-07

**Authors:** Per Milberg, Markus Franzen, Amanda Karpaty Wickbom, Sabine Svelander, Victor Johansson

**Affiliations:** ^1^ IFM Biology, Conservation Ecology Group Linköping University Linköping Sweden

**Keywords:** bees, bumblebees, flowering phenology, hoverflies, summer annual, Sweden, Syrphidae, vegetation, winter annual

## Abstract

The extent to which weeds in arable land are useful to pollinators depends in part on the temporal pattern of flowering and insect flight activity. We compiled citizen science data on 54 bees and hoverflies typical of agricultural areas in southern Sweden, as well as 24 flowering weed species classified as pollinator‐friendly in the sense that they provide nectar and/or pollen to pollinators. The flight periods of the bees and hoverflies varied greatly, but there were also some consistent differences between the four groups studied. The first group to fly were the early flying solitary bees (7 species), followed by the social bees (18 species). In contrast, other solitary bees (11 species) and hoverflies (22 species) flew later in the summer. Solitary bees had the shortest flight periods, while social bees and hoverflies had longer flight periods. Flowering of weed species also varied greatly between species, with weeds classified as winter annuals (e.g., germinating in autumn) starting early together with germination generalists (species that can germinate in both autumn and spring). Summer annuals (spring germinators) and perennials started flowering about a month later. Germination generalists had a much longer flowering period than the others. Weekly pollinator records were in most cases significantly explained by weed records. Apart from early flying solitary bees, all models showed strong positive relationships. The overall best explanatory variable was the total number of weeds, with a weight assigned to each species based on its potential as a nectar/pollen source. This suggests that agricultural weeds in Sweden provide a continuous potential supply of nectar and pollen throughout the flight season of most pollinators.

## Graphical abstract

The usefulness of weeds to pollinators depends partly on the temporal pattern of flowering and flight activity. We compiled citizen science data on observation of bees and hoverflies typical of agricultural areas in southern Sweden, as well as flowering of weed species classified as pollinator‐friendly. Flight periods among the 54 pollinator species varied greatly among species as did flowering among the weeds. Data suggest that agricultural weeds in Sweden provide a continuous supply of nectar and pollen throughout the flight season for all pollinators except early‐flying solitary bees.

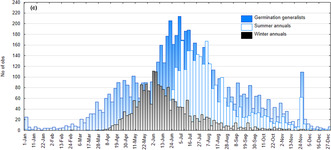

## 1 INTRODUCTION

The cultivation of cereals and other annual crops has undergone significant changes during the past two centuries, characterized by shifts in agricultural techniques and intensification processes (Shrubb, 2003; Tilman et al., 2011). The resulting land‐use changes have accelerated habitat loss and resource depletion for numerous organisms (e.g., Baude et al., 2016; Kleijn et al., 2015). One example is the loss of floral resources, which threatens pollinator populations and the ecosystem services they provide (Bartomeus et al., 2013, Carvell et al., 2006, Potts et al., 2010, Winfree et al., 2009), highlighting the urgency of addressing issues related to floral resource availability. Temporal variability in floral resources further exacerbates the challenge, as pollinators rely on consistent access to nectar and pollen throughout their foraging season (Ogilvie & Forrest, 2017; Rafferty & Ives, 2011; Timberlake et al., 2019).

The introduction of chemical pest control methods in the mid‐1950s revolutionized agricultural practices (Andreasen et al., 2018; Hyvönen et al., 2020), but also affected non‐target organisms and greatly reduced weed abundances in fields. Nevertheless, weeds remain a prevalent component of high‐input agricultural systems (e.g., Hald, 1999b, Milberg & Hallgren, 2004). Therefore, their ecological importance in agricultural landscapes cannot be ignored, as they provide habitat and food for various organisms, including pollinators, over large areas (Balfour & Ratnieks, 2022; Bretagnolle & Gaba, 2015; Chandrasena, 2022; Esposito et al., 2023; Nicholls & Altieri, 2013). Indeed, their relative importance for pollinators might have increased as the transition to intensified agricultural practices has also meant the loss of diverse habitats such as grasslands and marginal lands (Ammann et al., 2024; Brown & Schulte, 2011; Cousins et al., 2015; Fjellstad & Dramstad, 1999; Hietala‐Koivu, 1999).

Understanding plant‐pollinator relationships requires insights into the temporal patterns of flowering and flight activities (e.g., Kharouba et al., 2018; Milberg & Palm, 2024). Citizen science data appear to be particularly well‐suited to address such questions.

The aim of this study was to compare the flight pattern of pollinators that occur in agricultural landscapes in southern Sweden with the flowering of “pollinator‐friendly” weeds typical of arable land. The following hypotheses were tested:

1. The flight periods of hoverflies, social and solitary bees differ and hence also partly their temporal need for floral resources.
2. Flowering of weeds partly depends on the germination behavior of the species; e.g., winter and summer annuals flower at different times and provide floral resources for pollinator at different times.
3. Pollinator‐friendly weeds flower during most of the growth seasons, and flowering coincides with the flight of most pollinators.

## 2 MATERIALS AND METHODS

### 2.1 Citizen science data

Swedish citizen science data were used to compile information about flight periods of bees and hoverflies and flowering of weeds. Such data were downloaded from Artportalen (SLU, 2023) in April 2023 and included observational reports of species by both amateurs and professionals (currently >100,000,000 reports). The minimum information provided is species name, date and geographic position. Additional information potentially provided varies among taxa and involves development stage for insects (imago, larvae, etc) and plants (budding, flowering, fruiting, etc), and some type of quantification (number of specimens, number of shoots or area covered).

We limited our study area to Götaland in southern Sweden (latitude from 55 to 59) and to the time period 2008–2022 (as earlier years had fewer insect records). Throughout the current study, we combined observations without consideration of where within Götaland or type of habitat. Furthermore, we used the reported observation date as in‐data, irrespective of number of individuals reported by an observer.

For meaningful data on flight or flowering, the species had to be relatively frequently reported, and we arbitrarily decided only to include species with at least nine observations per year in at least 11 years out of 15 years included.

The onset and termination of flight are elusive features as they happen when population levels are at their smallest (Belitz et al., 2020; Van Strien et al., 2008). Consequently, they are highly sensitive to sampling effort, which complicates comparisons between species. In addition, if very early and very late observations are more likely to be reported than mid‐season observations, the flight season will be subject to observer bias. These issues are partly resolved if applying some arbitrary cutoff. We defined start of flight/flowering and the length of the flight/flowering period by excluding the first and last 10% of data, per species per year, which is in line with recent studies (Larsen et al., 2022).

### 2.2 Pollinators

The current study focused on pollinators occurring in arable‐dominated landscapes and hence that could potentially exploit weeds for nectar and/or pollen. The pollinators selected are known to fly within arable fields in Östergötland, according to a color pan trap study that caught >100 species of pollinators during July 2021 (P. Milberg, unpublished data). An additional requirement was that species should be reported as occurring in agricultural land and/or urban areas (www.artfakta.se; that among ecological information provides classification of landscape preference of species). We then excluded observations based on, e.g., larvae, pupae or dead individuals, and focused on reports of imago/adults and of observations of free‐flying or foraging individuals. Finally, we excluded rare species as described above (<9 observation in at least 11 of 15 years). These selection criteria resulted in a list of species (Table 1) that had 14 species of social bees (Apoidea), 7 species of early‐flying solitary bees, 11 species of summer‐flying solitary bees and 22 species of hoverflies (Syrphidae).

**TABLE 1:** Number of observations of the 24 weed species and the 54 pollinator species used in this study and their estimated start dates and lenghts of activity period.

	Number of observations	Start date (day number)	Flowering/flight period length (days)
WINTER ANNUALS			
*Buglossoides arvensis*	514	118.9 (8.7)	49.2 (22.5)
*Myosotis arvensis*	941	141.1 (7.3)	88.6 (21.6)
*Anthemis arvensis*	684	153.9 (5.9)	76.1 (22.6)
*SUMMER ANNUALS*			
*Sinapis arvensis*	485	156.7 (10.0)	121.3 (29.6)
*Erysimum cheiranthoides*	331	162.8 (10.2)	114.8 (20.7)
*Galeopsis speciosa*	461	180.1 (4.0)	54 (19.3)
*Fallopia convolvulus*	202	180.6 (8.6)	54.4 (18.9)
*Galeopsis tetrahit*	693	187.2 (4.1)	52.2 (19.9)
*Sonchus oleraceus*	515	190.9 (17.5)	102.5 (33.0)
*Galeopsis bifida*	430	192.5 (4.7)	53.7 (22.5)
GERMINATION GENERALISTS			
*Lamium purpureum*	1445	86.5 (20.5)	185.3 (45.4)
*Lamium hybridum*	445	108.5 (20.6)	176.9 (34.8)
*Lamium amplexicaule*	389	125.4 (15.3)	129.7 (24.8)
*Brassica napus* subsp, napus	199	141.8 (21.1)	112.5 (47.2)
*Anchusa arvensis*	745	155 (12.8)	113 (19.1)
*Centaurea cyanus*	1037	164.7 (8.9)	93.3 (21.1)
*Matricaria chamomilla*	573	164.8 (9.8)	79.2 (18.5)
*Tripleurospermum inodorum*	1327	167.2 (7.4)	113 (33.4)
*PERENNIALS*			
*Barbarea vulgaris*	892	125 (5.4)	28.7 (9.6)
*Ranunculus repens*	582	145 (5.8)	68 (32.5)
*Trifolium pratense*	1590	157.1 (4.6)	99.9 (26.8)
*Convolvulus arvense*	666	169.6 (9.1)	60.5 (11.6)
*Cirsium arvense*	897	179.1 (4.5)	60.9 (15.4)
*Sonchus arvensis*	850	186.5 (7.6)	67.7 (12.7)
SOCIAL APOIDEA			
*Apis mellifera*	828	85. (12.3)	137.6 (31.2)
*Bombus terrestris*	2220	90.8 (8.8)	126.9 (14.3)
*Bombus hypnorum*	1221	103.5 (6.1)	98.3 (8.9)
*Bombus pratorum*	1131	113.2 (7.5)	92.5 (10.5)
*Bombus lucorum*	1265	113.4 (19.8)	105 (21.9)
*Bombus lapidarius*	1540	119.5 (6.2)	100.4 (9.4)
*Bombus pascuorum*	1810	122.9 (8.6)	103.9 (13.0)
*Bombus bohemicus*	381	130.9 (12.5)	81.8 (14.2)
*Bombus hortorum*	491	141.9 (15.7)	65.8 (17.7)
*Bombus sylvarum*	450	153.6 (24.5)	66.3 (24.9)
*Bombus rupestris*	341	155.1 (8.4)	59.1 (20.0)
*Bombus soroeensis*	375	159.4 (19.5)	61.1 (18.9)
*Bombus subterraneus*	266	167.1 (10.9)	33.7 (18.4)
*Bombus humilis*	204	172.3 (21.0)	40.2 (22.1)
SOLITARY APOIDEA, spring‐flying			
*Andrena vaga*	241	94.3 (13.1)	26.1 (16.3)
*Andrena clarkella*	210	96.3 (6.8)	21.6 (8.8)
*Andrena fulva*	644	105.2 (5.4)	26.7 (10.2)
*Osmia bicornis*	292	110.3 (7.8)	37.3 (16.3)
*Andrena nigroaenea*	270	111.9 (10.2)	40.6 (15.8)
*Andrena haemorrhoa*	482	112.7 (4.8)	40.3 (12.9)
*Andrena cineraria*	214	115.1 (9.2)	34.1 (20.1)
SOLITARY APOIDEA, summer‐flying			
*Sphecodes albilabris*	189	121.9 (21.1)	95.4 (33.6)
*Eucera longicornis*	344	148.5 (5.1)	30.7 (10.4)
*Andrena hattorfiana*	1623	173.1 (5.5)	31.8 (4.6)
*Cerceris rybyensis*	279	173.9 (10.5)	35.1 (16.3)
*Bembix rostrata*	204	182.6 (12.2)	29.7 (11.1)
*Megachile lagopoda*	412	184.1 (13.8)	20.1 (12.4)
*Philanthus triangulum*	561	185.3 (16.7)	35.5 (12.6)
*Dasypoda hirtipes*	369	192.2 (7.0)	27.3 (11.4)
*Panurgus calcaratus*	231	197.3 (10.8)	23.4 (12.9)
*Andrena denticulata*	191	200.2 (10.7)	21.8 (8.6)
*Andrena marginata*	435	208.9 (10.0)	27.5 (10.3)
*SYRPHIDAE*			
*Rhingia campestris*	204	139.8 (20.6)	61.9 (31.3)
*Merodon equestris*	202	142.5 (30.6)	40.1 (33.5)
*Helophilus pendulus*	819	145.1 (8.7)	114.5 (15.3)
*Eristalis intricaria*	436	146.6 (20.9)	81.7 (19.3)
*Eristalis interrupta*	235	149 (18.5)	75.5 (20.9)
*Myathropa florea*	542	151.1 (8.2)	62.3 (10.6)
*Eristalis pertinax*	540	152.5 (20.9)	110.7 (32.9)
*Xylota segnis*	230	155.1 (7.4)	48.1 (14.4)
*Volucella bombylans*	299	156.1 (6.1)	42.1 (11.0)
*Eristalis tenax*	508	157.2 (43.4)	121.5 (45.8)
*Syrphus ribesii*	256	157.9 (26.6)	84.1 (37.1)
*Syritta pipiens*	349	161.5 (19.4)	77.7 (24.9)
*Eupeodes corollae*	336	163.9 (15.9)	53.1 (15.2)
*Chrysotoxum festivum*	169	166.5 (9.7)	46.3 (23.9)
*Volucella pellucens*	694	169.7 (7.6)	48.8 (4.8)
*Eristalis arbustorum*	201	165.3 (24.3)	69.7 (24.4)
*Sericomyia silentis*	544	164.3 (9.1)	74.8 (20.9)
*Sphaerophoria scripta*	363	164.3 (20.2)	73.9 (22.7)
*Episyrphus balteatus*	1151	173.5 (15.1)	80.1 (28.0)
*Helophilus trivittatus*	202	175.9 (23.6)	60.2 (20.2)
*Scaeva pyrastri*	372	179.4 (15.3)	42.7 (22.5)
*Leucozona glaucia*	211	190.0 (11.9)	27.9 (14.7)

*Note*: Estimated start date and length of activity were calculated after eliminating the first and last 10% of data, per species per year. Numbers within parenthesis are SD, based on 15 years of data. The weed species are known to provide floral resources to pollinators, and pollinator species are known to search arable fields for food. Data from Götaland in southern Sweden.

### 2.3 Weeds

Two criteria were used to select the weed species: First, they should be widespread and relatively common within arable fields in southern Sweden. Data used to assess this criterion were prevalence in Swedish weed control trials (Hallgren, 1996, P. Milberg, K‐O. Bergman, L. Björklund & L. Westerberg, unpublished). Second, species should be potentially relevant for pollinators, i.e., providing either nectar or pollen in some quantity. For this assessment, we used a recent classification of Swedish plant species (Tyler et al., 2021). Here, plant species are classified into one of seven different classes reflecting their importance for pollinators, but among the potential weeds only six of the classes were represented. Two potential weed species were missing from Tyler et al. (2021) and were therefore assigned values according to the same criteria using other sources: *Taraxacum* coll. (6; Baude et al., 2016) and *Fumaria officinalis* (3; Ouvrard & Jacquemart, 2018). We selected weeds scoring ≥4 and defined them as “pollinator‐friendly weeds”; 4: nectar production modest (5–20 g sugar/m^2^/year); 5: rather large (20–50 g); 6: large (50–200 g; Tyler et al., 2021).

The 24 weed species selected (Table 1) were then classified as perennials or annuals, the latter further divided into winter and summer annuals (i.e., species that germinate predominantly in autumn and spring, respectively), as well as “germination generalists” (i.e., species that can germinate in both autumn and spring). This classification was based mainly on Swedish sources (Fogelfors, 2006, 2022; Milberg et al., 2000), to ensure regional relevance, but also one general source (Hanf, 1984).

We only included reports of flowering individuals, excluding non‐flowering development stages as well as the many reports that did not report development stage.

### 2.4 Statistics

To test the difference in flight start between the four pollinator groups, we modeled the day of flight start each year in relation to pollinator group using a generalized mixed effect model (GLMM) with a normal distribution and year as random effect. We used the same model structure to analyze the length of the flight period for the four pollinator groups, as well as the flowering start of the four weed groups and the length of their flowering period.

To test the relationship between pollinator records and weed flowering, we used weekly counts of the different groups of pollinators and weeds. We then modeled the weekly records of each pollinator group (and all pollinator groups summed together) in relation to each group of weeds using a GLMM with a negative binomial distribution (over‐dispersed counts) and year as random effect. We also tested the weekly total records of weeds (all groups summed together) and an index weighting the importance of different weed species for pollinators when summed together (Tyler et al., 2021), as explanatory variables. The explanatory power of each variable was then assessed based on the decrease in AIC (∆AIC) compared to the null model.

## 3 RESULTS

### 3.1 Pollinators

**FIGURE 1:**
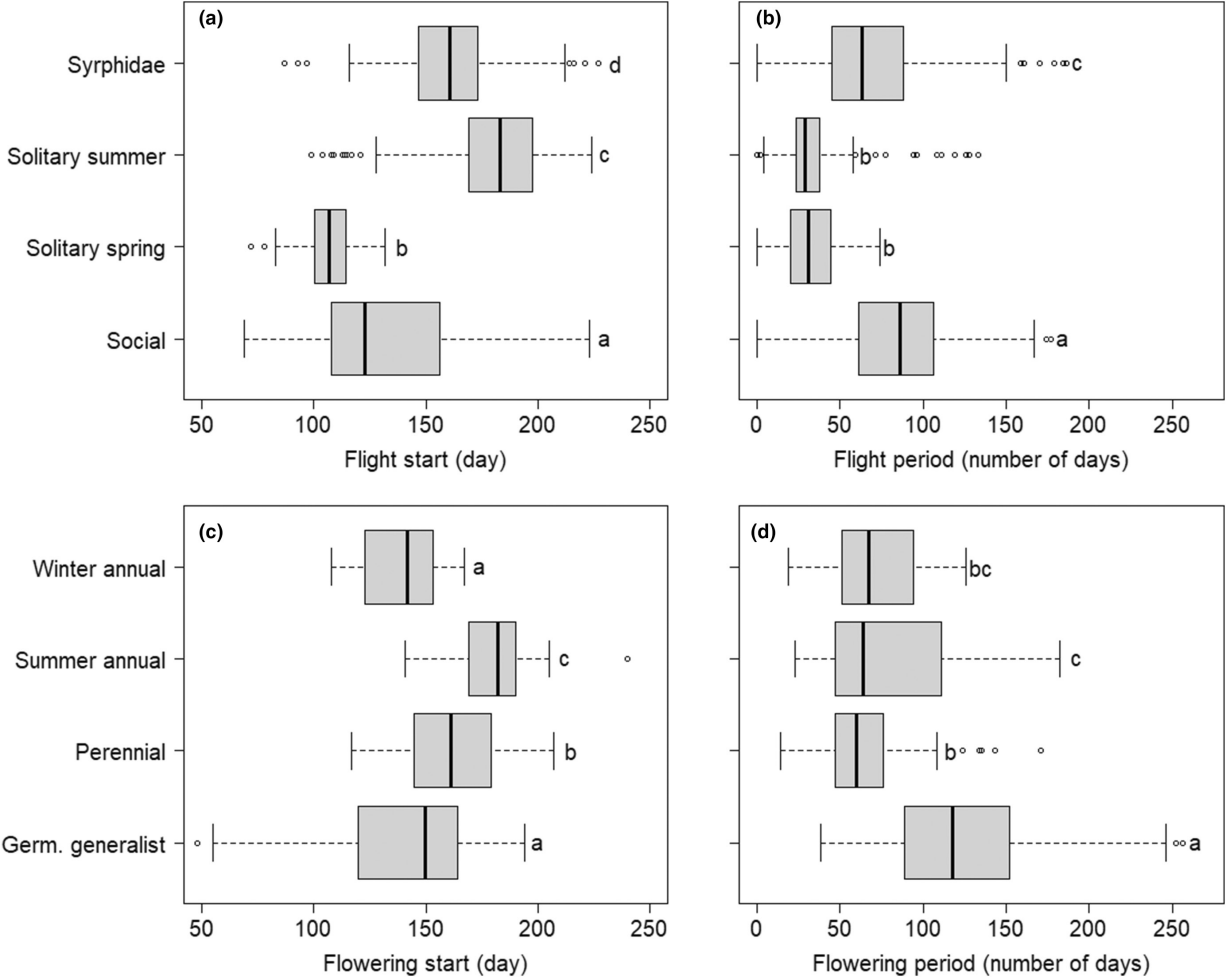
Flight start and flight period of pollinators occurring in agricultural landscapes and flowering start and flowering period of weeds of arable land that are potential nectar/pollen sources. Citizen science data based on 15 years from the province of Götaland, southern Sweden, with the first and last 10% of records eliminated per year.

The timing of flight and the length of flight periods varied greatly among the 54 species (Table 1). Overall, however, early‐flying solitary bee species and social bees had the earliest flight starts (Figure 1a). Species of hoverflies and social bees had the longest flight periods while solitary bee species had the shortest flight period (Figure 1b).

The longest flight periods recorded for a species, defined as 80% of the observations per year, were *Apis mellifera* (138 days), *Bombus terrestris* (127), *Eristalis tenax* (122), *Helophilus pendulus* (114) and *Eristalis pertinax* (111; Table 1). The shortest flight periods were among solitary bees, and especially the genus *Andrena* where four species had a flight period of less than 25 days (Table 1).

**FIGURE 2:**
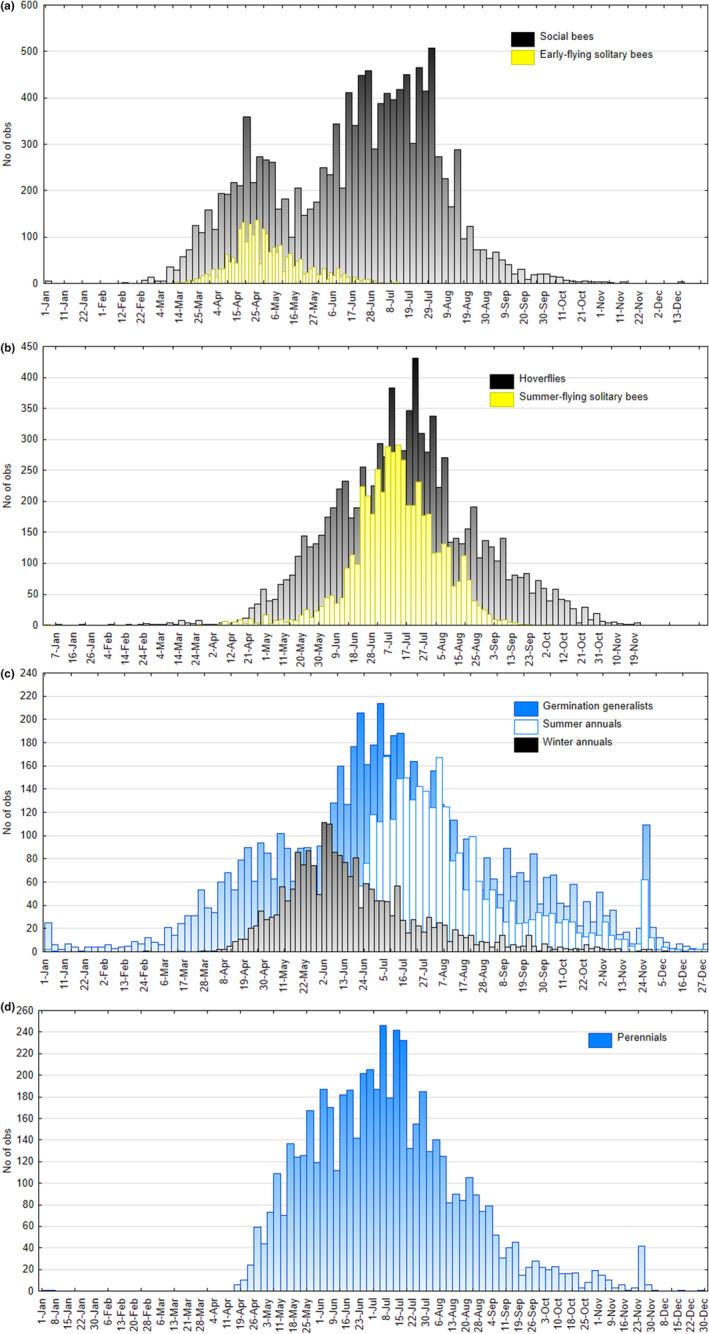
The temporal pattern of all observations of pollinators occurring in agricultural landscapes and flowering of weed species of arable land that are potential nectar/pollen sources. Data summarize 15 years of observations from the province of Götaland, southern Sweden.

The temporal distribution of observations revealed that social bees demonstrated a pronounced bimodal activity pattern, with many observations in late April followed by a dip during May and a major peak in mid‐July (Figure 2a).

### 3.2 Flowering of weeds

Flowering among the weeds also varied greatly among species (Table 1). Weeds classified as winter annuals and germination generalists started flowering first (mid‐May), while summer annuals flowered about a month later (Figure 1c). Germination generalists had the longest flowering period (Figure 1d), Most species flowered for 2–3 months (Table 1). The germination generalist *Lamium* spp. flowered for more than 4 months (130–185 days), while the perennial *Barbarea vulgaris* had the shortest flowering period (29 days; Table 1). When considering the temporal pattern of reports of flowering weeds, there were many reports (>200 per week) from late April to early October and a peak around early July (Figure 2).

### 3.3 Temporal patterns of weed flowering and pollinator records

Weekly pollinator records were in most cases significantly explained by weed records (Table 2). With the exception of spring‐flying solitary bees, all models yielded positive relationships, and often highly significant ones (Table 2). Hence, the flowering of weeds coincided with the flight of all pollinator groups except the early‐flying solitary bees (Figure 2). All pollinator groups (except spring‐flying solitary bees) and the total count of pollinators was best explained by the total weed counts (social bees) or the index where weeds were weighted based on their potential to produce pollen/nectar (summer‐flying solitary bees, hoverflies, and total count, Table 2).

**TABLE 2:** The parameter estimates (with SE) and *p*‐values for the models of weekly pollinator counts in relation to different weed groups.

		Social bees	Spring‐flying solitary bees	Summer‐flying solitary bees	Hoverflies	All pollinators
Germination generalist	Par. Est (SE)	0.88 (0.07)***	−0.31 (0.15)*	0.92 (0.11)***	0.92 (0.08)***	1.26 (0.09)***
	∆AIC	157.8	2	84.2	168.7	248.2
Perennial	Par. Est (SE)	0.67 (0.08)***	−0.53 (0.10)***	1.20 (0.10)***	1.10 (0.07)***	0.92 (0.07)***
	∆AIC	125.3	22.4	120.3	275.8	202.8
Summer annual	Par. Est (SE)	0.45 (0.06)***	**−1.04 (0.11)** ***	1.18 (0.10)***	0.83 (0.07)	0.65 (0.07)***
	∆AIC	61.6	**75.8**	152	164.2	109.4
Winter annual	Par. Est (SE)	0.66 (0.09)***	−0.07 (0.10)	0.23 (0.11)*	0.62 (0.08)***	0.84 (0.09)***
	∆AIC	81.7	−1.4	2.7	68.1	109.3
Total count of flowers	Par. Est (SE)	**0.78 (0.08)** ***	−0.65 (0.12)***	1.31 (0.09)***	1.16 (0.12)***	**1.14 (0.08)** ***
	∆AIC	**159.1**	23.6	167.5	295.1	**258.4**
Total count of flowers weighted by index	Par. Est (SE)	0.76 (0.06)***	−0.71 (0.11)***	**1.34 (0.09)** ***	**1.17 (0.07)** ***	1.10 (0.08)***
	∆AIC	152.9	30.8	**179.7**	**299.9**	247.7

*Note*: ΔAIC = change in AIC when adding the variable to the Null model. The best model (largest ΔAIC) is shown in bold.

* *p* < .05.

*** *p* < .001.

## 4 DISCUSSION

This study used around 45,000 observations to calculate estimates of flowering times of weeds on arable land and flight times of pollinators in agricultural landscapes. The aim was to compare these two groups to assess the potential value to pollinators of floral resources within arable fields. It is important to note that the observations were spread across different habitats and that pollinators exploit a landscape made up of a mosaic of different habitats. Among them, the interior of arable fields is a neglected habitat that dominates in terms of acreage.

### 4.1 Flight of pollinators

A striking feature in the data was the considerable variation among pollinator species regarding both onset and length of flight (cf Pawlikowski et al., 2020; Westrich, 1990). There were, however, also clear differences among the four groups of pollinators considered. As expected, social bees, including the honeybee, had the longest flight periods and were also among the first to commence their flight (cf Bartomeus et al., 2011; Pawlikowski et al., 2020), while the two types of solitary bee species often flew for a short period in southern Sweden; this confirms that few solitary bees in Sweden are bi‐ or polyvoltine. Hoverflies, on the other hand, commenced their flight later in the season—mid‐June—with long flight periods extending into September and even October (Owen, 1981).

The data for social bees displayed a clear bimodal phase of activity with a peak in April, followed by a mid‐May decrease and then a large peak in July. This could be interpreted as an effect of queens' flights early and then followed by a buildup of worker number until it peaks in July, as shown in previous studies (Goodwin, 1995; Gurel et al., 2008; Teräs, 1976). Unfortunately, sex was very rarely reported in our data (<0.9%) which precludes any test of this explanation. An alternative explanation for the bimodal patterns might be a reduced flight activity of bumblebees during a “June gap” in floral resources that has been documented elsewhere (Jachuła et al., 2021; Timberlake et al., 2019). In some areas, it seems to be caused by a superabundance of food by a mass‐flowering crop in May followed by much lower food availability during June (Jachuła et al., 2021; Requier et al., 2015). We are not aware of any reports of such a gap in Sweden and note that a corresponding “May gap” seems unlikely, given that is when the only mass‐flowering crop in the study area flowers: autumn‐sown rape. During our study period, rape covered 6.7% or the arable land and was mainly made up of autumn‐sown types (95%; SOS, 2024). Hence, the dip in bee activity in May coincided with the flowering of rape. Could mass‐flowering rape cause a substantial shift in flight patterns of bumblebees, given that arable land is only one of several habitats available? Members of nests close to rape fields might fly much shorter distances and would hence be less likely to be observed. On the other hand, members of other nests might make longer flights to reach rape fields. So, on balance, we believe that the observed dip in bumblebee activity in May is unrelated to food shortage similar to the “June gap” phenomenon documented elsewhere. Instead, we find the most likely explanation being a succession from bumblebee queen to worker foraging trips.

A long flight period can be caused by a long lifespan of imagos, the degree of synchrony of hatching among individuals, and by a species having more than one generation within growth seasons. Such bi‐ or polyvoltine behavior is common among hoverflies (Speight, 2008; Terry & Nelson, 2017; Wratten et al., 1995), among which some species also migrate to Sweden (Bartsch et al., 2009; Bartsch & Binkiewicz, 2009), explaining the long flight periods recorded for many species. In fact, at least 8 of the 22 Syrphid species studied showed a bimodal distribution of observation dates (with minimum numbers in May, June, July or August; data not shown), suggesting a bivoltine behavior. In contrast, only one species among the solitary bees, was a candidate for bivoltine behavior using the same criteria: *Sphecodes albilabris* had a surprisingly long flight period with two distinct flight peaks (early May and mid‐August; data not shown). However, the dual peaks are the result of over‐wintering adult females; hence, this species too is univoltine (Westrich, 1990). To conclude, the short flight period recorded in the current study confirms the conclusion that almost all solitary bees are univoltine in Sweden (Linkowski et al., 2004). Social bees make up a special case with non‐parasitic species having long‐lived queens, and several overlapping generations of workers (Ogilvie et al., 2017) and hence, as expected, long flight periods as recorded in our observational data. Even the two parasitic bumblebees in the current study had long flight periods (*Bombus bohemicus*, *B. rupestris*).

Consequently, judging by the flight activity recorded in citizen science data (Figure 2), the full assemblage of pollinators in Sweden's agricultural landscapes would require floral resources from early April to mid‐September.

### 4.2 Flowering of pollinator‐friendly weeds

The weed species studied had all been considered as potentially useful for pollinators (Tyler et al., 2021). Species differed in both onset and length of flowering. Here too, the functional groups showed some consistent differences, mainly that flowering of winter annuals started a month before summer annuals, results consistent with Hirose et al. (2005), Håkansson (1983, 1995) and Fogelfors (2006). In contrast to further south in Europe, many annual weed species in Sweden do not fit well in the winter/summer annual dichotomy (e.g., Karlsson & Milberg, 2007, 2008), and we had therefore defined a third category: “germination generalists” that can germinate in both autumn and spring. Most species in this group had very long flowering periods, probably due to cohorts of germinants emerging in different seasons. The long flowering time of this group of species suggests they might be particularly valuable pollinators in arable land.

Perennials are often considered as more important than annuals for pollinators (Hicks et al., 2016). On the other hand, annuals invest a larger proportion of their biomass to sexual reproduction than perennial species (Albani & Coupland, 2010) and make up much more biomass on arable land (Andersson & Milberg, 1998; Salonen et al., 2023), which points to the importance of annuals.

It is worth noting that the data used reflect the flowering in all types of habitats. If we want to assess the importance of these plant species on arable land, we need also to consider the ways in which crop management interfere with flowering. First, sowing time of crops is a critical factor and the trend towards more autumn‐sown crops (Hald, 1999a) suggests an increasing importance of winter annuals and germination generalists and a declining importance of summer annuals. Even the precise sowing date can be important for the weed flora developing (Huusela‐Veistola et al., 2006; Milberg et al., 2001). We hypothesize that winter annuals and germination generalists that have germinated in the autumn are particularly important for the early flight of social and solitary bees. Second, harvest and the preparation for sowing that follows means an early termination of flowering of summer annuals and germination generalists during the first half of August (when harvest normally happens in Sweden). Hence, late‐flying species, like many hoverflies, should find limited floral resources on arable land in the latter part of their flight season, a period during which floral abundance is generally decreasing (Balfour et al., 2018; Fitzpatrick et al., 2007; Garbuzov et al., 2020; Guezen & Forrest, 2021; Timberlake et al., 2021).

It is also worth pointing out that as weed flowering continues unabated during summer and the rest of the growth season (Figure 2), flowering in Swedish grasslands decreases from 1 July (Roth et al., 2023). This suggests that the relative importance of floral resources on arable land increases compared with alternative habitat, at least up to harvest.

To summarize, the weed species studied have the potential to support pollinators for the full flight season, with the exception of early‐flying solitary bees. If one limits the assessment to plants on arable land, harvest terminates flowering, leaving parts of August and September as a period with limited floral resources on arable land, and this would mainly affect late‐flying hoverfly species.

Autumn‐sown crops normally have more weed biomass; for example, in 1053 weed control experiment in autumn‐sown cereals in Sweden, the average fresh weight of weeds in treated plots was 40 g/m^2^ while corresponding estimate for spring‐sown cereals (918 experiments) was 20 g/m^2^ (P. Milberg & L. Westerberg, unpublished data). In untreated plots, corresponding values were 329 and 211 g/m^2^ (Milberg et al., 2000). This biomass difference is likely due to longer time allowed for germination and growth as well as differences in weed species composition. These biomass differences are likely mirrored by floral resources being larger and more long‐lasting in autumn‐sown crops. Notable is also that autumn‐sown crops provide floral resources early in the season, when annuals in spring‐sown areas just have germinated but not yet started flowering. We hypothesize that a shift towards more autumn‐sown acreage might assist social bees (flight of queens) and those solitary bees that fly early.

On a methodological note, we used reports of all pollinator‐friendly weeds, as well as an index where the numbers were weighted by the “pollinator index” of each species (in our case 4, 5 or 6). Often, the latter better reflected the pollinators recorded, suggesting a way to improve the usefulness of the flowering data that is often included in pollinator studies (e.g., Ammann et al., 2024; Guezen & Forrest, 2021; Mallinger et al., 2016; Scheper et al., 2015).

### 4.3 Citizen science data

The current study used 28,577 and 16,893 observations of 54 pollinator and 24 weed species over 15 years, respectively. Collecting such data is beyond the scope of most research projects, pointing to the great potential of Citizen data. Still, there are some limitations in the data used worth pointing out and that are rarely considered in studies using, e.g., GBIF data (e.g., Duchenne et al., 2020).

First, the identification of organisms can be a challenge and missed occurrences cannot be avoided in Citizen science data (Kremen et al., 2011; Ratnieks et al., 2016). The species included in the current study, however, are relatively easy to identify, so misidentification is not a major concern.

Second, and more importantly, observations reflect when and where observers take notes. The spatial bias, i.e., that some areas or habitats are more frequently visited, is a major limitation in some type of studies using Citizen Science data requiring complex data analyses (e.g., Bradter et al., 2018; Snäll et al., 2011), but for the phenological issue under scrutiny here it is unlikely to undermine the conclusions (Rzanny et al., 2024). More important for the present study is a temporal bias in reports where some periods involve much more field activity by observers. We believe that insect and plant observations in the autumn are generally affected by a negative bias, while the opposite is likely in spring and early summer. Unusual times, like winter observation of flowering or honeybees or hoverflies, are also more likely to be over‐reported. Another surprising find in our study was the many reports of flowering from late November. Was this a date reporting error, or was someone particularly searching for late flowering? These potential biases involving early and late reports, justified using the arbitrarily defined flight period as the period from 10% to 90% of the observations. This caveat should be kept in mind when evaluating our data, and the potential risk of early period of pollen‐shortage predating the flight period. The main reason for truncating the flight records, however, was to eliminate the bias in flight start and length caused by number of observations.

On balance, the type of citizen science data used here seems particularly useful to evaluate phenological changes due to weather and climate, but only for more frequently reported species.

## 5 CONCLUSION

This study has shown that the total flight period of pollinators in agricultural landscapes is long, partly accentuated by differences among bees and hoverflies. Furthermore, agricultural weeds in Sweden provide a continuous potential supply of nectar and pollen throughout the flight season for all but the earliest flying solitary bees. Early floral supply—important for social bees and some solitary bees—is likely most pronounced in autumn‐sown crops, while harvest eliminates late floral supply, mainly affecting hoverflies.

## AUTHOR CONTRIBUTIONS

**Per Milberg:** Conceptualization (equal); formal analysis (equal); investigation (equal); methodology (equal); project administration (equal); supervision (equal); writing – original draft (equal). **Markus Franzen:** Methodology (equal); resources (equal); writing – review and editing (equal). **Amanda Karpaty Wickbom:** Data curation (equal); formal analysis (equal); investigation (equal); writing – original draft (supporting). **Sabine Svelander:** Data curation (equal); formal analysis (equal); investigation (equal); writing – original draft (supporting). **Victor Johansson:** Formal analysis (lead); visualization (equal); writing – review and editing (equal).

## CONFLICT OF INTEREST STATEMENT

The authors have no conflicts of interest to declare.

## Data Availability

The data used here have been extracted from www.artportalen.se where anyone can download data. We slightly edited data, as described in Methods. The actual data used in analyses are available on request.
